
JOURNAL INFORMATION
==============================
NLM Title Abbreviation: Atlanta Med Surg J (1884)
NLM Journal ID: 101251623

Atlanta Medical and Surgical Journal (1884)

Publisher: Biomedical Journal Digitization Project

ARTICLE INFORMATION
==============================
PMCID: PMC9201863
PMID: 35829611
Article version: 1
Subjects: Society Reports

Regular Meeting of the Atlanta Society of Medicine

Print publication date: 1899 Feb
Volume: 15
Volume Series: New Ser
Issue: 12
First page: 815
Last page: 821
License: This work is free of known copyright restrictions. For more information, please see PMC Back Issue Digitization.
License URL: https://creativecommons.org/publicdomain/mark/1.0/

==============================

### SOCIETY REPORTS.

### REGULAR MEETING OF THE ATLANTA SOCIETY OF MEDICINE.

Atlanta, Ga., January 19, 1899.

The meeting was called to order by the President, Dr. W. S. Goldsmith.

The following members were present: Drs. Champion, Childs, Crawford, Duncan, Goldsmith, Hardon, Hutchins, Kime, G. P. Robinson, Dunbar Roy, W. Monroe Smith, Claude A. Smith, Stirling, VanGoidtsnoven.

The minutes of the previous meeting were read and approved.

The application for membership of Dr. C. C. Geer was read and referred to the Executive Committee.

Dr. W. Monroe Smith read a paper upon "Treatment of Abortion."

### DISCUSSION.

*Dr. Hardon:* There is very little left for me to say, as I think Dr. Smith has covered the ground admirably, and his views coincide with my own. In case of premature expulsion of the ovum, I think that there is no other treatment after the abortion becomes inevitable than the complete dilatation of the cervix and emptying of the womb. This, of course, means an operation, but it is entirely devoid of danger if properly and thoroughly done. If it is not properly done there is a great deal of danger; but since any operation or procedure is to be judged only by the results when properly done, therefore I think it is the most satisfactory treatment. It has several advantages. In the first place, as Dr. Smith has said, it saves time, not for the physician, but for the patient, and lessens her suspense and anxiety. As Dr. Smith says, the plan of tamponing the vagina involves a delay of from twelve to twenty-four hours if we get suitable results, and this is doubtful.

If we control the hemorrhage thoroughly we must pack the vagina to its utmost; and the first time a physician tries it he is astonished at the amount of cotton necessary, as from one to two pounds will be needed to absolutely pack the vagina. This amount left for twenty-four hours gives more pain than arises from the abortion. Another objection is that it is not easy to do it thoroughly, as it will often happen that the blood will escape and the hemorrhage go on. These are serious objections; the pain and delay are serious, and that we cannot be sure of controlling the hemorrhage is equally serious.

By dilating the cervix and thoroughly removing the contents we know when it is empty. Unless we explore the entire womb we can never be sure that it is completely empty. I make it a rule never to treat a case of abortion without exploring with my finger and finding whether it is thoroughly emptied. At the same time, if we are going to pass the finger into the uterus, it is very little more of an operation to pass in the curette and curette thoroughly, and I irrigate at the same time the curetting is going on. I have often read that the best instrument for emptying the womb is the finger, but I have not found this to be so. It is hard to grasp fragments with the fingers and you are apt to do damage. I prefer the double curette. In regard to the antiseptic solution, I always use the bichloride solution. I have seen only one case in which there was unpleasant results from the use of the bichloride and it was then used in the strength of 1–2000th. It should not be used so strong. I use 1-5000th, and have never seen any trouble with this. I compliment Dr. Smith upon the excellence of his paper.

*Dr. Stirling:* This question is hardly one in which I have any special interest, but I can remember having the pleasure of attending a good many of these cases. I arise to ask a question. In those days it seemed the difficulty was that the cervix would not dilate, but it would close and you could not tell whether the fetus was expelled or not. I have heard lately that cocain applied would open it, and I arise to ask if this is the experience of any one here?

*Dr. Duncan:* I don't think I have anything to add to what has been said. Dr. Smith says he prefers morphine as a nerve sedative; I think we all do that, but I would not discard the viburnum prunifolium. It has acted well with me, and have not had the nausea which he mentions. In regard to the curette, I have had great difficulty, as Dr. Hardon says, in removing the ovum with the finger, but I prefer to use this as I am not familiar with the use of the curette. I have succeeded with the finger in every instance, although it is difficult in some cases. I have not had the experience with the curette that others have had and I hesitate to use it. The dull curette I consider safe but the sharp curette I do not, and the finger is perfectly safe when properly used. The plan of dilating the cervix is the correct thing when the abortion is inevitable.

*Dr. Kime:* This is a subject in which all practitioners are interested. I was very much interested in the paper and, in the main, agree with it, but I cannot say that I agree that morphia is the best to prevent the expulsion of the fetus. The combination which has given me the best success is codeine, bromides and liquor sedans. When I fail with this then I empty the uterus. The objection that I have to morphia is that it makes them sick and constipates the bowels, but with the other you get not only the nerve sedative of the bromides but you get the narcotic effect of the viburnum. I cannot say that I fully agree on the question of tamponing the vagina to prevent hemorrhage. My experience has not been as unfortunate as that of the doctors who have spoken. However, I do not advocate it as routine work. Here in the city we are in easy reach of all the necessary instruments, etc., for dilating the cervix and curetting, but it is not so with the majority of the country and general practitioners. When you get out eight or ten miles into the country you have to resort to something else when you do not have the instruments with you. In cases in which the abortion is inevitable I do not think anything is better than the tamponing. I take a strip of gauze and carry it up into the cervix and then put in a small amount of cotton. I do not pack the vagina full, and have never used more than a quarter of a pound, but have always controlled the hemorrhage with this.

As to the question of using the curette or the finger, I have found that I could do better with the instrument than with the finger. I have found the ordinary sponge-holding forceps excellent to remove any particles and then I curette the placental site.

As to irrigation, I have been in the habit of using the bichloride solution, and I have not seen any trouble following it, as there is very little risk in a solution of 1-4000, which is the strength I use. I have always used a little camphorated phenol for 24 to 48 hours, and this acts as a stimulant as well as an antiseptic. While speaking on the line of infection, I would say that I believe that 90 per cent. of infections are of putrid infection and not septic infection after abortion.

Two little instruments have been called to my attention for removing the contents of the uterus after abortion, and I think they are better for the general practitioner than the curette. I have used them in several cases and have had good results, except in one or two cases where there was abnormal condition of the uterus. I have not been in the habit of using the irrigator curette, as it interferes with the delicacy of touch.

*Dr. W. Monroe Smith* (closing the discussion): In regard to the use of cocain to dilate the cervix, I have never had that suggested before and have never used it in my practice. I have used the dilators, which act on the same principle as a pair of forceps and which is introduced into the cervix and then by pressing the handles the blades are separated.

I heartily agree with the method of passing the finger into the uterus to be sure that it is empty, and I have found it a good precaution. I have used the liquor sedans with very nice results and do not remember a case with nausea, but I have had nausea in two-thirds of the cases where I have used simply the viburnum, and as nausea is a frequent cause of abortion, I have avoided using it. Codeine is good, and where I find the nausea in some people, I then use the codeine, but my experience with it has been that it constipates the bowels about as badly as any other preparation of opium.

The President stated that Dr. Butler would be unable to be present to read his paper upon "Traumatic Aneurism," but that

Dr. G. P. Robinson would read a paper upon "Footwear of Children."

DISCUSSION.

*Dr. Hardon:* I have nothing to say about shoes, but would like to relate an incident. Yesterday I was in the house of a physician and his wife spoke about suffering a great deal with her feet. I glanced down at her foot and saw that she wore a long narrow shoe, more like a tooth-pick. I called her attention to the shoe, but it was impossible to convince her that her trouble was due to the shoes, and I suppose she will go on wearing such shoes. It convinced me as much as any one case could convince one. We see cases often and I hope the paper of Dr. Robinson will lead to good results.

*Dr. Stirling:* I have nothing to say except to thank Dr. Robinson for his kindness in informing us where we can obtain these common-sense shoes, and am glad that one store will have them.

*Dr. Hutchins:* This paper is something that is very interesting for one who has to buy shoes for children. Some people's feet are naturally easy to fit and they can get a shoe that is comfortable from the start. I have found that I can get a shoe in the store that will fit me better than to have one made. In the case of my little girls, one of them is very easily fitted and it is no trouble to get a shoe that is comfortable and fits perfectly, while with the other one, it is difficult to get a comfortable shoe. As regards the appearance and shape of shoes, people go by the custom and not by what is best for them.

*Dr. Kime:* This is a question in which we are all interested, especially if we have corns. I have long since adopted a common-sense shoe, but in my younger days I did not have quite such good judgment. In children it is the formative stage and they should be fitted properly then. I think this is a step in the right direction, but so long as it is the fashion to wear a sharp-pointed toe, it is hard to get the people to wear a sensible shoe; but it is not so hard to start the fashion for children, and we should direct our attention to this.

*Dr. Van Goidtsnoven:* I arise to thank Dr. Robinson for his paper, and hope he will have it published.

*Dr. Duncan:* I think the paper is very opportune if children will wear shoes, but I think the parents should think of how much happier and better off they would be if they did not wear shoes until seven or eight years of age. They would have less colds and would be healthier every way. When they wear shoes they get their feet wet, and let them stay wet, and thus they take cold. I knew a preacher who would not put shoes on his children until they were four or five years of age. It was remarkable how well they kept, but after this age every one had colds. I really believe that the wearing of shoes is injurious to children, not only from the shape of the shoes, but by keeping them off the ground.

*Dr. Robinson:* I think it would be a good thing to let the children run bare-foot sometime, but it would be rather bad this time of the year.

The reason I brought up this paper was on account of the influence the physicians might exert over the mothers. Mothers will do almost anything for their children, and if you convince them of the injurious effects it will not be difficult to get them to change. The people in New York and Boston are taking a sensible view of this matter, and are wearing only the broad-toed shoe. The dealers cannot sell these narrow-toed shoes. All that is necessary is a little missionary work on the part of each one of the profession.

RELATION OF CASES.

*Dr. Crawford:* A case of some interest came before me some two or three weeks ago. It was a tooth in the left nostril. The party was about 33 years of age, and came to me with some nose trouble. I examined the nose and found a granular mass in the floor of the left nostril. I put a little cocaine on it and took a scalpel and prized it open and found an ivory looking substance, and decided it was a tooth. I had never seen such a thing in that position before, and I showed it to some dentists, and they found that I was correct. With my bistoury I cut through the granular mass, and with a pair of artery forceps, was able to lift the tooth out. It was nearly the original size, and it was driven from its natural position into the nose. He said that some fourteen years ago, he had fallen against the mantel, and struck his tooth and knocked it out, but could not find it. One point of interest was the force that was necessary to drive this tooth through the small socket and up into the nostril. Evidently this had given him a great deal of trouble. Perhaps he did not notice the condition very much, but he said, however, that he had gone to a specialist some ten years before for this trouble, but failed to detect this tooth then. The root of the tooth was in perfect order, but the body was more or less decayed or broken off.

*Dr. Hutchins:* A case was brought to me last week from the country by a doctor, and he and every one believed it to be malignant. The patient had extracted a left molar, wisdom tooth, and there grew from this a soft sessil growth. The doctor had been burning this down and it grew back very rapidly. It was supposed to be a sarcoma. I broke off a piece of the growth very easily and had it mounted, and I would say that I got all sorts of malignant diagnoses, which illustrates how hard it is to diagnose a sarcoma under the microscope without the clinical history. At first glance under the microscope, it looked as if it was a small, round-celled sarcoma. There were all kinds of growth of the mucous membrane. I simply, finally, made a diagnosis of polypoid growth of the gum, and curetted thoroughly, and I think the patient will get well. There was no evidence, clinically, of sarcoma. One thing that seemed to impress the doctors in the country was that the patient had had a polypoid growth in the cheek, and they were inclined to attribute this growth to the one inside.

Another case I was undecided whether it was gumma of the tongue or sarcoma. I have not yet diagnosed it. The patient has a large ulcer which has been cultivated by the use of nitrate of silver. The patient objected to an operation, and under the use of iodides, etc., is getting better. However, the case is still *sub judice.* The iodide is being used as an aid to diagnosis. If it was a gumma, of course would expect more than one. It is a very muched mixed up case.
